# Recurrent Lactobacillus Rhamnoses Bacteremia and Complications in an Immunocompromised Patient With History of Probiotic Use: A Case Report

**DOI:** 10.7759/cureus.54879

**Published:** 2024-02-25

**Authors:** Ujunwa J Eze, Anthony Lal, Menatallah I Elkoush, Marta Halytska, Syed Atif

**Affiliations:** 1 Family Medicine, WellSpan Good Samaritan Hospital, Lebanon, USA

**Keywords:** lactobacillus infections, lactobacillus bacteremia–associated morbidity, immunosuppression, probiotics, lactobacillus rhamnoses bacteremia

## Abstract

The use of probiotics has been on the rise in the past few years. Increasing reports on Lactobacillus bacteremia-associated morbidity and mortality in immunocompromised patients have raised safety concerns about its use in this group. We present a case of a 79-year-old patient with a history of probiotic use who was admitted from the emergency department for acute-on-chronic diarrhea, acute hyponatremia, acute-on-chronic diastolic heart failure, and ambulatory dysfunction. His multiple co-morbidities include stage III chronic kidney disease, type II diabetes mellitus, chronic diastolic heart failure, Parkinson’s disease, essential hypertension, atrial fibrillation, status post pacemaker placement, and status post bioprosthetic aortic valve replacement. He was diagnosed with a resistant case of Lactobacillus bacteremia during his admission, with multiple positive blood cultures positive for L. rhamnosus resistant to antibiotics therapy. Unfortunately, due to multiple complications related to Lactobacillus bacteremia, he transitioned to comfort care and ultimately passed away in a few weeks. Although probiotics are generally considered safe, their safety in immunocompromised patients is uncertain. Until more research is available to confirm their safety, caution should be taken when using them in this population. This study is an addendum to the few studies on this topic.

## Introduction

Over the years, probiotics have been shown to be effective in the prevention and treatment of various medical disorders, especially disorders of the gastrointestinal tract [1]. Lactobacillus rhamnosus GG (LGG), being the first probiotic, has achieved the most clinical relevance to date. Since probiotics are available over the counter, patients do not often discuss with their healthcare providers before starting probiotics. Although recent studies have shown increasing use of probiotics among hospitalized patients, the prevalence of Lactobacillus bacteremia is still low [2-4]. However, it is more common in individuals taking probiotics, especially immunocompromised patients [3,5]. Lactobacillus species, being a normal body flora, has generally not posed much concern about causing disease in immunocompetent individuals. In fact, they are considered contaminants or opportunistic pathogens when isolated from specimens belonging to immunocompetent individuals [6]. Increasing reports on Lactobacillus bacteremia-associated morbidity and mortality in immunocompromised patients have raised concerns about the safety of probiotic use in this group [5-8]. Interestingly, the direct causes of the mortality and morbidity are rarely the disease itself but rather from complications of the disease or from other comorbidities [3]. We report a unique and rare case of a 79-year-old immunocompromised male with Lactobacillus rhamnosus bacteremia.

## Case presentation

A 79-year-old patient was admitted for acute-on-chronic diarrhea, acute hyponatremia, acute-on-chronic diastolic heart failure, and ambulatory dysfunction. He presented with diarrhea and generalized weakness. His chronic medical problems included previous Lactobacillus bacteremia and endocarditis about five months prior to presentation, for which he was treated with daptomycin and penicillin G, chronic diastolic heart failure, chronic diarrhea (for which he was taking probiotics), Parkinson's disease, stage III chronic kidney disease (CKD), diabetes mellitus (DM) type II, essential hypertension, chronic anemia, atrial fibrillation, status post pacemaker placement, and status post bioprosthetic aortic valve replacement (AVR).

On admission, vital signs were normal; physical examination was significant for generalized weakness, ambulatory dysfunction, bilateral lower extremity pitting edema, and a new systolic heart murmur. Laboratory workup showed acute hyponatremia, elevated brain natriuretic peptide (BNP), and elevated C-reactive protein (CRP). CT scan of the head without contrast was normal. Chest X-ray showed cardiomegaly, right basilar atelectasis, pleural fluid, and consolidative changes in the lung base. His CT of the abdomen showed small free fluid and atherosclerotic disease. While his diarrhea was being worked up, his blood culture came back positive for gram-positive rods suspected to be L. rhamnosus due to previous history of L. rhamnosus bacteremia. He was started on empiric antibiotics with Unasyn® and vancomycin pending further maturation of blood culture. A transthoracic echocardiogram (TTE) was done following a positive blood culture. This came out negative for obvious vegetation, warranting follow-up with a transesophageal echocardiogram (TEE) due to high suspicion in a high-risk patient (status post AVR and pacemaker). His blood culture matured to confirm the suspicion of L. rhamnosus. 

Subsequently, he had multiple positive blood cultures for L. rhamnosus; he was continued on IV Unasyn® and vancomycin until susceptibility testing came out showing Lactobacillus bacteremia strain was susceptible to penicillin; hence, vancomycin was discontinued. 

TEE was suggestive of possible aortic root abscess next to the prosthetic valve. Possible management options, including surgical and conservative management, were discussed with the patient and family. After some deliberations and getting a second opinion from a different cardiologist, the patient was deemed high risk and, hence, not a good candidate for surgical intervention.

**Figure 1:**
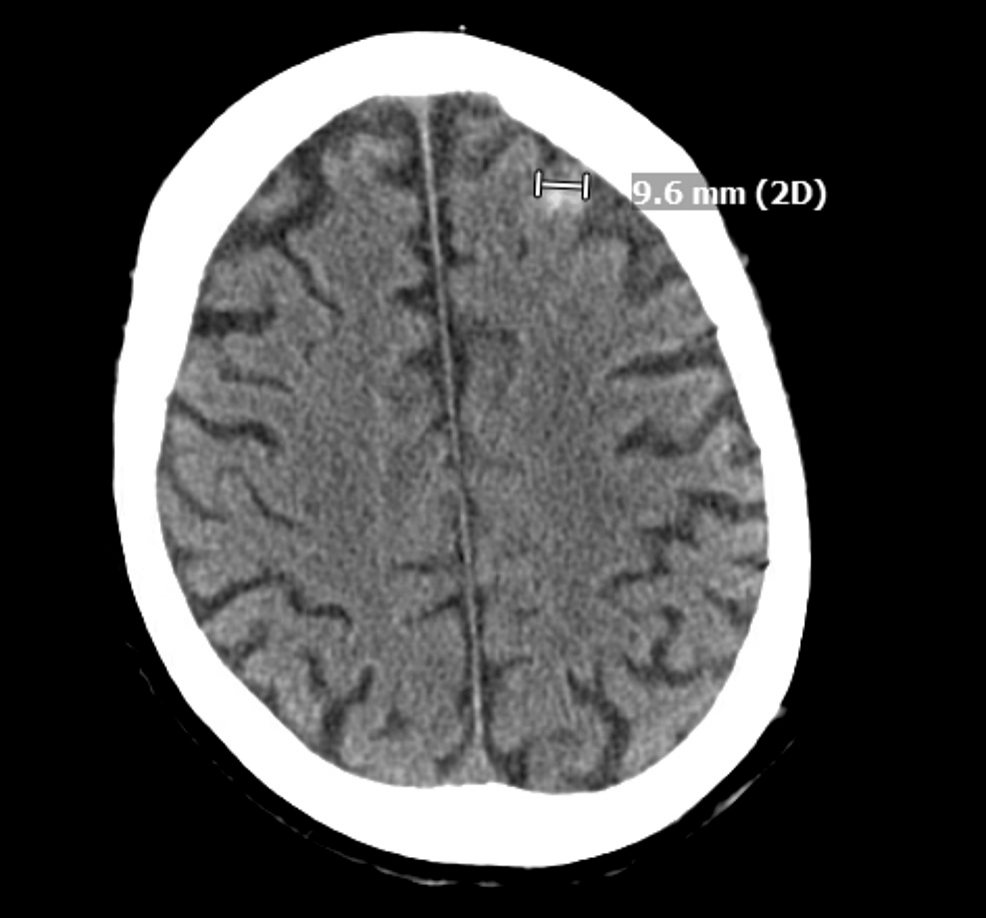
CT scan of the head following stroke incident showing a focal area of left frontal acute parenchymal hemorrhage

The hospital course was complicated by anemia, acute thrombocytopenia, acute on chronic kidney disease, acute on chronic heart failure (CHF), hypokalemia, stroke concerning septic emboli from the heart, Candida glabrata-related urinary tract infection (see Table 1 and Figure 1).

**Table 1:** Trend of hemoglobin, platelets count, and white blood cells counts during hospital admission

Date	10-Jan-2023	12-Jan-2023	13-Jan-2023	14-Jan-2023	15-Jan-2023	16-Jan-2023	17-Jan-2023	18-Jan-2023	19-Jan-2023	20-Jan-2023	21-Jan-2023	22-Jan-2023	23-Jan-2023	24-Jan-2023	25-Jan-2023	26-Jan-2023	27-Jan-2023	28-Jan-2023	29-Jan-2023	29-Jan-2023	30-Jan-2023	31-Jan-2023	1-Feb-2023	2-Feb-2023	3-Feb-2023	4-Feb-2023	5-Feb-2023	6-Feb-2023	7-Feb-2023
Time	5:02 PM	5:59 AM	5:40 AM	5:05 AM	5:11 AM	6:41 AM	4:28 AM	3:40 AM	3:42 AM	3:42 AM	3:49 AM	5:15 AM	3:36 AM	4:03 AM	2:53 AM	3:16 AM	3:36 AM	6:01 AM	7:36 AM	6:02 PM	2:45 AM	4:25 AM	4:58 AM	2:17 AM	7:20 AM	3:34 AM	5:14 AM	4:37 AM	5:54 AM
Hemoglobin (latest reference range: 13.0-17.3 g/dL)	11.5	10.5	10.7	10	10.6	10.5	9.7	10.3	10.1	9.7	9.1	10	9.6	9.3	9.1	8.6	9.2	8.2	8.2	9	7.9	7.9	7.6	7.8	7.9	8	8.1	7.7	7.7
Platelet count (latest reference range: 140-400 K/mcL)	174	146	126	111	105	99	89	98	88	110	97	111	115	109	121	117	142	127	134	139	131	142	126	133	142	160	153	130	124
White blood cells (latest reference range: 4.0-11.0 K/mcL)	7.3	5.6	6.3	5.5	5.8	5.9	5.1	7.7	5	6.8	8.3	7.9	8.2	7.3	6.6	6.5	6.7	4.8	4.9	4.8	5.8	6.4	8.1	7.3	7.8	7.7	6.3	7	5.6

 

 

 

 

 

 

At some point, the patient was severely fluid-overloaded from CHF exacerbation, and acute kidney injury (AKI) worsened with diuresis. We continued aggressive therapy per the patient and family's decision while palliative continued to follow up. About halfway into his admission, the patient had a hemorrhagic stroke with symptoms of dysarthria, two episodes of tonic-clonic seizure, and absence seizures necessitating transfer to ICU. According to neurology assessment, this could be related to an embolic source from septic emboli, given his possible endocarditis/bacteremia in the setting of bio-prosthetic valve replacement and pacemaker placement. Despite aggressive treatment and management of numerous hospital complications, the patient continued to have an overall decline in function.

We requested different consulting services, including infectious disease, cardiology, nephrology, neurology, occupational and physical therapy, speech-language pathology, nutrition, social workers, chaplain pastoral care, and palliative care. Following multiple family meetings with the palliative team, he was transitioned to comfort care and discharged to a hospice care facility. He passed away a few weeks later.

## Discussion

Lactobacillus spp. are a normal body flora known to inhabit the oral cavity, gastrointestinal tract, and genital tract, where they protect these organs from colonization by pathologic bacteria - colonization resistance mechanism [9,10]. Probiotics are widely used in over-the-counter diets and are known to protect against and treat various medical conditions [1]. In immunocompetent individuals, these organisms are not expected to cause any disease condition. However, in immunocompromised individuals, these organisms have caused infections ranging from localized infections to bacteremia [10,11]. Lactobacillus can cause bacteremia by damaging the intestinal barrier, mucosal damage, immunosuppression, abnormal changes in the intestinal microbiome, and visceral ischemia [12]. Although Lactobacillus infections are majorly endogenous in origin, they can also have exogenous causes [5,10-12]. There have been increasing reports of episodes of Lactobacillus bacteremia in the last 20 years, which are widely attributed to the increasing use of probiotics [4,12]. Immunosuppression is the single most important risk factor for the development of Lactobacillus bacteremia. Most immunosuppression was related to cancer, diabetes, solid organ transplant, hospitalization, comorbidities [2,3,10]. Our patient had diabetes and CKD, disorders that can cause significant immunosuppression. Although most Lactobacillus bacteremia cases are immunocompromised individuals, there are still reported cases of Lactobacillus infections in immunocompetent individuals [8,11].

Successful treatments for these patients require a careful choice of antimicrobial therapy. First, a positive culture and susceptibility testing are of utmost importance in the treatment of Lactobacillus bacteremia [11]. Also, combination antibiotic therapy with penicillin and aminoglycoside is the widely recommended treatment for these patients [5]. Although the antimicrobial susceptibility of clinical Lactobacillus blood isolates in patients with Lactobacillus bacteremia characterized at the species level showed that most of the clinical Lactobacillus isolates showed low minimum inhibitory concentration for imipenem, piperacillin-tazobactam, erythromycin, and clindamycin but had variable susceptibility to penicillin and cephalosporins [7,13]. Despite the narrow susceptibility options, microbiologically guided treatment has been shown to improve survival in patients with Lactobacillus bacteremia [11,13].

Antimicrobial treatment of Lactobacillus bacteremia can be challenging, especially in patients with multiple morbidities, such as ours [10,11]. This could be due to multiple factors, including polypharmacy with increased risk of drug-drug interaction, contraindications to antibiotics due to other morbidities, complications of bacteremia, and variable susceptibilities to antibiotics [7]. For instance, in patients with AKI, aminoglycoside is contraindicated due to its nephrotoxicity. Our patient, during the first occurrence of Lactobacillus bacteremia with possible endocarditis, was treated with IV penicillin G with daptomycin. When he presented with a reoccurrence of Lactobacillus bacteremia, we initially started him on broad-spectrum antibiotics, including IV ampicillin/sulbactam and vancomycin, pending sensitivity results. Vancomycin was stopped due to patients developing AKI, plus sensitivity results showed susceptibility to penicillin. Furthermore, persistent (or recurrent) bacteremia with Lactobacillus is not uncommon. This can be attributed to inadequate treatment, probably due to the above-mentioned challenges. Recurrent bacteremia has also been attributed to delayed identification [14].

Like other causes of bacteremia, Lactobacillus bacteremia can lead to several complications. Some of these complications could be from the seeding of bacteria in the bloodstream into various organs or sepsis with end-organ damage. During his admission, our patient developed multiple complications that made management challenging. He developed AKI, which limited our antimicrobial therapy and diuretics use in the setting of chronic heart failure. An aortic root abscess was found on TEE. Of note, surgical intervention plays a vital role in the management of aortic root abscess, which can be very fatal and has high mortality and morbidity if not diagnosed early and treated with the prompt surgical intervention [6]. However, surgical intervention was not recommended due to our patient having an increased risk of mortality owing to many comorbidities and a history of prosthetic aortic valve. The patient also developed bilateral knee pain, likely due to septic arthritis, though enough samples for culture could not be obtained from arthrocentesis to confirm this diagnosis. The patient developed acute thrombocytopenia, for which we held medications, including aspirin and allopurinol. About halfway into his hospital stay, he developed acute-on-chronic dysarthria, difficulty finding speech, two episodes of tonic-clonic seizure, and absence seizures. CT scan of the head showed evidence of hemorrhagic stroke, suspicious of thromboembolic stroke. Furthermore, withholding Eliquis® was recommended for the patient, which left him at increased risk for more thromboembolic events. In addition to these complications, the patient had other complications that affected treatment options, including anemia, hypoalbuminemia, and electrolyte imbalances.

Mortality in patients with Lactobacillus bacteremia has been estimated at a level of 30%, with a direct cause of death not necessarily from bacteremia [3]. In most cases, the cause of death is from underlying conditions or complications of Lactobacillus bacteremia [3,10]. Several studies have reported mortality despite appropriate antibiotic treatment. In an earlier study, mortality of Lactobacillus bacteremia was found to be 26% at one month after onset of illness and 48% at one year after onset of illness [11]. A high mortality rate of 69% one year after onset of illness has been reported in another study [5]. Our patient died two weeks after transitioning to palliative care despite treatment with penicillin, which the Lactobacillus isolates were susceptible to.

## Conclusions

Our study adds to the increasing number of reported cases of Lactobacillus bacteremia, especially in immunocompromised individuals. This study also brings to light that most times, the direct cause of mortality and morbidity from Lactobacillus bacteremia is rarely from the disease itself but rather from the complications of the disease or from other comorbidities. Hence, although probiotics are generally considered safe, their safety in immunocompromised patients is uncertain. Until more research is available to confirm their safety, caution should be taken when using them in this population.
